# Repetitive Behaviours in Patients with Gilles de la Tourette Syndrome: Tics, Compulsions, or Both?

**DOI:** 10.1371/journal.pone.0012959

**Published:** 2010-09-24

**Authors:** Yulia Worbe, Luc Mallet, Jean-Louis Golmard, Cécile Béhar, Franck Durif, Isabelle Jalenques, Philippe Damier, Pascal Derkinderen, Pierre Pollak, Mathieu Anheim, Emannuel Broussolle, Jing Xie, Valérie Mesnage, Karl Mondon, François Viallet, Pierre Jedynak, Mouna Ben Djebara, Michael Schüpbach, Antoine Pelissolo, Marie Vidailhet, Yves Agid, Jean-Luc Houeto, Andreas Hartmann

**Affiliations:** 1 Centre d'Investigation Clinique INSERM CIC 9503, Pôle des Maladies du Système Nerveux, Assistance Publique-Hôpitaux de Paris, Groupe Hospitalier Pitié-Salpêtrière, Université Pierre et Marie Curie-Paris 6, Paris, France; 2 Département de Biostatistique, Assistance Publique-Hôpitaux de Paris, Groupe Hospitalier Pitié-Salpêtrière, Paris, France; 3 Département de Neurologie, Centre Hospitalo-Universitaire de Clermont-Ferrand, Clermont-Ferrand, France; 4 CHU Clermont-Ferrand, Service de Psychiatrie de l'Adulte A et Psychologie Médicale, Pôle de Psychiatrie, Clermont-Ferrand, France; 5 Clermont Université, Université d'Auvergne Clermont 1, UFR Médecine, Equipe d'Accueil 3845, Clermont-Ferrand, France; 6 Département de Neurologie, Centre Hospitalo-Universitaire de Nantes, Nantes, France; 7 Département de Neurologie, Centre Hospitalo-Universitaire de Grenoble, Grenoble, France; 8 Université Claude Bernard Lyon I, Faculté de Médecine Lyon-Sud Charles Mérieux, Hospices Civils de Lyon, Département de Neurologie C, Hôpital Neurologique Pierre Wertheimer, Centre de Neurosciences Cognitives, CNRS UMR 5229, Lyon, France; 9 Département de Neurologie, Centre Hospitalo-Universitaire de Poitiers, Poitiers, France; 10 Département de Neurologie, Centre Hospitalo-Universitaire de Tours, Tours, France; 11 Département de Neurologie, Centre Hospitalier d'Aix-en-Provence, Aix-en-Provence, France; 12 Département de Psychiatrie, Assistance Publique-Hôpitaux de Paris, Groupe Hospitalier Pitié-Salpêtrière, Paris, France; CNRS, France

## Abstract

**Background:**

Repetitive behaviours (RB) in patients with Gilles de la Tourette syndrome (GTS) are frequent. However, a controversy persists whether they are manifestations of obssessive-compulsive disorder (OCD) or correspond to complex tics.

**Methods:**

166 consecutive patients with GTS aged 15–68 years were recruited and submitted to extensive neurological, psychiatric and psychological evaluations. RB were evaluated by the YBOCS symptom checklist and Mini International Neuropsychiatric Interview (M.I.N.I), and classified on the basis of a semi-directive psychiatric interview as compulsions or tics.

**Results:**

RB were present in 64.4% of patients with GTS (107/166) and categorised into 3 major groups: a ‘tic-like’ group (24.3%–40/166) characterised by RB such as touching, counting, ‘just right’ and symmetry searching; an ‘OCD-like’ group (20.5%–34/166) with washing and checking rituals; and a ‘mixed’ group (13.2%–22/166) with both ‘tics-like’ and ‘OCD-like’ types of RB present in the same patient. In 6.3% of patients, RB could not be classified into any of these groups and were thus considered ‘undetermined’.

**Conclusions:**

The results confirm the phenomenological heterogeneity of RB in GTS patients and allows to distinguish two types: tic-like behaviours which are very likely an integral part of GTS; and OCD-like behaviours, which can be considered as a comorbid condition of GTS and were correlated with higher score of complex tics, neuroleptic and SSRIs treatment frequency and less successful socio-professional adaptation. We suggest that a meticulous semiological analysis of RB in GTS patients will help to tailor treatment and allow to better classify patients for future pathophysiologic studies.

**Trial Registration:**

ClinicalTrials.gov NCT00169351

## Introduction

Gilles de la Tourette syndrome (GTS) is a neurodevelopmental disorders characterised by the presence of motor and vocal tics [1]. Tics are brief, stereotypical, repetitive and non-rhythmic movements or phonations that typically follow a waxing and waning pattern of severity, intensity and frequency [1], [2]. Tics can be differentiated from other hyperkinetic movement disorders by the possibility of their temporary voluntary suppression. They are also often anticipated by premonitory sensations or ‘urge to do’, relieved by performance of the tic [1], [2].

GTS and obsessive-compulsive disorder (OCD) share several characteristics: both disorders have a juvenile or young adult onset, a chronic waxing and waning course and are characterised by the presence of repetitive behaviours (RB) and premonitory urges [3], [4]. OCD is characterised by repetitive, intrusive thoughts and images and/or by repetitive, ritualistic physical or mental acts performed to reduce anxiety [5]. The cognitive model of OCD suggests that the intrusive cognition serves for the OCD patients as an indication that they are responsible for harm and its prevention [6]. Consequently, patients with OCD experience anxiety discomfort (egodystonic character of intrusive cognition) and are engaged in anxiety-neutralizing compulsive behaviours [4].

When tics are present in patients with OCD, the patients are predominantly males, have an earlier age of symptoms onset [7], [8] and a higher frequency of sensory phenomena preceding RB [9], [10]; repetitive thoughts and behaviours are mostly characterised by religious obsessions or cleaning, washing or counting compulsions [11]–[13]. In contrast, when OCD is diagnosed in patients with GTS, repetitive thoughts and behaviours are frequently characterised by intrusive violent and sexual images or thoughts, as well as hoarding, counting and symmetry searching rituals or touching behaviour [11], [12].

Based on comparative studies of RB in patients with GTS and those with ‘pure’ OCD, some authors argue that RB and thoughts in patients with GTS are non-anxiety-related, have an egosyntonic (personally comfortable, without pre-existent anxious state) character [4] and are performed in a stereotypical or automatical way [12]–[15]. The terms ‘impulsions’ or ‘tic-like compulsions’ were proposed for these types of RB [12]–[14], [16]. However, other reports have failed to show differences in obsessional beliefs between ‘pure’ OCD and patients with GTS with RB [7], [17]. Consequently, it remains unclear whether the repetitive behaviours and thoughts in patients with GTS are performed to reduce anxiety due to obsessional beliefs as veritable compulsions, or as tic-like behaviours initiated by premonitory sensations or ‘urge to do’.

The present study was designed to categorise RB in a large group of consecutive patients with GTS by classifying them into compulsions or tics; and to verify whether the different types of RB influence the clinical expression of GTS. We hypothesized that RB in patients suffering from GTS (i) represent more likely tic-like behaviours and (ii) do not share the same semiological features as RB observed tic-free OCD (hand-washing, “folie du doute”). As a consequence, we suggest that patients with GTS could benefit from better treatment adaptation if RB are correctly categorised.

## Methods

### Subjects

The study was performed on adolescent (minimum age: 15 years) and adult patients with GTS. All patients were recruited via movement disorders university centers between January 2005 and November 2008. The study was promoted by the Institut National de la Santé et de la Recherche Médicale (INSERM) and approved by the Medical Ethical Committee of the Pitié-Salpêtrière Hospital, Paris, France. All participants gave written informed consent; for minors, written informed consent was obtained from the parents/guardians.

The inclusion criteria were DSM-IV diagnosis of GTS, with the ability to give written informed consent. The exclusion criteria were evaluated by administering the French version of Mini International Neuropsychiatric Interview (M.I.N.I; version 5.0.0) [18] to all patients by trained psychologists or psychiatrists before entering the study. Patients suffering from concomitant psychosis, current major depressive episode, autistic spectrum disorders, substance abuse (except nicotine) or mental deficiency were excluded from the study. Patients who refused to complete the interview assessment (n = 7) or suffering from tics associated with other types of movement disorders (n = 4) were excluded from the study as well.

### Clinical assessment

The clinical assessment of patients was performed by movement disorders neurologists and psychiatrists experienced in GTS and OCD. The general medical history as well as the GTS history was collected for all patients included in the study. Simple and complex motor and vocal tics were carefully checked by the neurologist in every patient. Complex tics were scored by the sum of all complex tics (vocal and motor) presented in each patient to avoid confusion with RB. The severity of tics was rated using the tic portion of Yale Global Tic Severity Scale (YGTSS) [19]. The symptoms' impact on social and occupational functioning was evaluated using the ‘Overall Impairment’ item of the YGTSS and the ‘Global Functioning Evaluation Scale’ (GAF) [20].

The presence, type and duration of RB and thoughts were identified with the YBOCS symptom checklist [21] (which identifies both OCD and RB close to tics, and is frequently used in genetic studies on both disorders) and the M.I.N.I version 5.0.0 [18]. In accordance with DSM-IV criteria of OCD, if *all* repetitive behaviours combined and thoughts lasted less than one hour per day, they were not included in the final analysis.

To distinguish compulsions and obsessions from ‘tic-like’ behaviours and thoughts, a semi-directive psychiatric interview was developed by two psychiatrists (L. M. and A. P.). The rationale was to obtain an expert psychiatric opinion on the presence of obsessions or compulsions in a standardised format and to exclude other psychiatric conditions in relation with ruminative thoughts or stereotyped behaviours (File S1). To standardize the evaluation among the experts, the psychiatrists evaluating patients across all french centres received training how to use this interview appropriately prior to the beginning of the study.

Following this semi-directive interview, repetitive behaviours and thoughts were defined as compulsions and obsessions if they (1) had clear egodystonic nature, i.e. experienced as personally uncomfortable, unwanted and senseless [22], (2) were performed to reduce pre-existent anxiety or to protect from real or potential harm, (3) produced important distress or augmentation of pre-existent anxiety when attempted to be suppressed or delayed, (4) and if the patient felt responsible of the potential negative events that could occur due to non-realisation of the ritual.

Repetitive behaviours and thoughts were defined as ‘tic-like’ behaviours or thoughts if (1) the patients had the need to perform behaviour similar to a tic as an ‘urge to do’ or in response to a premonitory urge; (2) they could be temporarily suppressed or delayed; (3) they were not directly associated with an anxious mood nor with the wish to control a risk of damage. It should be noted that active tic suppression can cause a build-up of inner tension which has not to be confused with anxiety/distress as experienced when compulsions are attempted to be suppressed. Thus, the major criteria to distinguish compulsions from tics were (i) the existence of anxiety before realisation of RB and (ii) RB being experienced as personally uncomfortable, unwanted and senseless. Nevertheless, RB had to respond to all criteria to be classified as obsession, compulsion or tic-like.

Every type of RB or thought was independently examined by neurologists and psychiatrists and referred to phenomenological groups as follows: (i) group of compulsions and obsessions (‘OCD-like’ group); (ii) group of ‘tic-like’ RB and thoughts). The RB or thoughts were classified as undetermined if both criteria for tics and compulsions or obsessions were identified for the same RB, or if the RB did not correspond to all criteria of compulsions or tics. The final phenomenological classification was performed on the basis of agreement between the evaluators.

Two types of RB were scored but not evaluated as criteria for tics or compulsions: (i) echophenomena and coprophenomena as they are considered as highly specific to GTS and have been suggested to be a predictive factor of the syndrome [4]; (ii) self-injurious behaviours (SIBs) that are suggested to be heterogeneous in the phenomenological expression and genesis, sharing characteristics of both stereotyped behaviours and compulsions [2], [23], [24].

### Statistical analysis

Descriptive statistics for clinical characteristics of patient's population and groups used numbers and frequencies for qualitative variables. The quantitative variables were characterised by the mean value ± standard deviation and by the range. Group characteristic comparisons were performed using chi-squared tests for qualitative variables, and ANOVAs followed by Tukey's tests for quantitative variables, allowing multiple pair-wise comparisons.

The equality of the distributions of the types of RB between ‘OCD-related’ and ‘tic-related’ groups was first tested using a maximum likelihood ratio test between two embedded log-linear models, namely one model with equal frequencies for all RB types, and another one with one frequency for each RB type. In a second step, frequencies of each RB type were compared between ‘OCD-related’ and ‘tic-related’ groups by chi-squared tests. This method can be viewed as a protection method for multiple comparisons.

The null hypothesis was rejected at a p-value <5% and all statistical tests were two-sided. Computations were performed using the SAS V8 statistical package.

## Results

### Patients

Clinical characteristics of patients and their treatments are provided in Table S1.

Different types of RB were identified in 64.5% (107/166) of patients with GTS. The comparative analysis showed that patients with RB expressed more frequently self-injurious behaviours compared to non-RB patients (p = 0.01), whereas we did not find a statistically significant difference in expression of echolalia (p = 0.6), echopraxia (p = 0.85) or coprophenomena (p = 0.3) between patients with GTS with and without RB.

### Classification of patients with GTS with repetitive behaviours

On the basis of the semi-directive interview and neurological assessment, all patients with RB were referred to four quantitatively non-equal (p = 0.0002) distinct phenomenological groups. 24.3% of patients (40/166) had phenomenological characteristics compatible with the criteria of tics – the ‘tic-like’ group. In 20.5% of patients (34/166), these corresponded to the criteria of compulsions and obsessions and were referred to the ‘OCD-like’group. In 13.2% (22/166) of patients with, several types of RB were present in the same patient corresponding to tic criteria for some and compulsions for others. Consequently, these patients were referred to as the ‘mixed’ group. Finally, in 6.3% (11/166) of patients, these did not correspond to all criteria of compulsions or of tics or, conversely, included both of them. These types of RB were classified as ‘undetermined’ and were not considered in the comparative analysis of the groups.

The three principal groups of GTS patient with RB (‘tic-like’, ‘OCD-like’ and ‘mixed’ group) had similar age, age of syndrome onset as well as the score of tic portion of YGTSS (Table S2). Nevertheless, all patients with RB had a higher score of complex tics (measured by the score of sum of all complex tics) compared to the group of non-RB patients (p = 0.01 for the ‘tic-like’ group; p = 0.05 for the ‘OCD-like’ group and p = 0. 0003 for the mixed group; Table S2). The ‘OCD-like’ group and the mixed group had the highest score for complex tics of all of groups. There were no statistically significant differences for echophenomena, coprophenomena and SIBs between the groups of patients with RB (Table S2).

Comparative analysis of treatment showed that the non-RB patients received the least treatment by neuroleptics (p = 0.02) amongst all groups of patients. An increase in neuroleptic medication was noted in the ‘tic-like’ group, followed by the ‘OCD-like’ group. Finally and by far, the mixed RB group had the highest treatment rate by neuroleptics (Table S2).

The frequency of treatment by selective serotonin reuptake inhibitors (SSRIs) was also different among the groups: the mixed and ‘OCD-like’ groups received more frequently SSRIs treatment compared to those who belonged to the ‘tic-like’ group or non-RB patients (p = 0.003, Table S2).

The scores for the overall impairment item of the YGTSS and the GAF reflected the treatment rates by neuroleptics and SSRIs: the ‘OCD-like’ group (p = 0.03 for both items) and the mixed group (p = 0.03 for GAF) had higher scores compared to the non-RB patients, whereas no differences were found in the ‘tic-like’ group compared to non-RB patients (p = 0.08 and p = 0.6, respectively).

### Different types of repetitive behaviours in patients with GTS

According to the YBOCS symptom checklist [21], all RB in patients with GTS were classified as follows: checking, washing, ordering and counting rituals, hoarding, symmetry searching, touching behaviour, phobias and ‘just right’ phenomena. Rare RB in our patients, such as repetitive smelling or praying, were referred to as ‘other rituals’.

The most frequently observed types of RB in patients with GTS were: touching in 78.5% (84/107), counting in 54.2% (58/107), symmetry searching in 33.6% (36/107) and ‘just right phenomena’ in 45.8% (49/107). Checking rituals were identified in 30.8% (33/107) and washing rituals in 10.3% (11/107) of our population of patients with RB.

**Figure 1:**
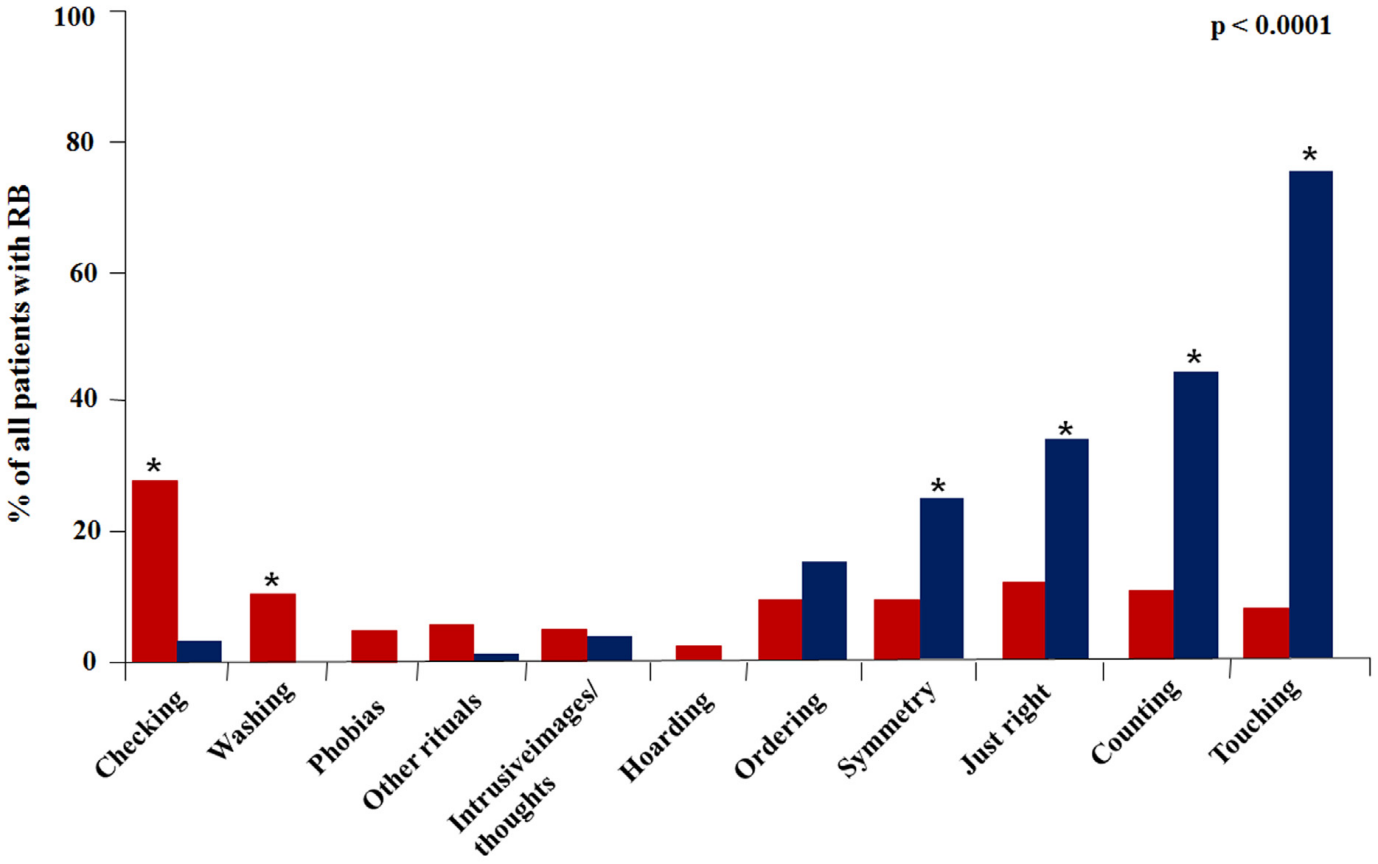
Percentage of different types of repetitive behaviours in our GTS patients sample. Red columns: ‘OCD-like’ RB; blue columns: ‘tic-like’ RB. * p<0.0001.

Detailed analysis revealed that different types of RB were distributed unequally between two principal phenomenological - ‘tic-related’ and ‘OCD-like’ groups (p<0.0001) (Figure 1). The RB that strongly corresponded to tic criteria were: touching (p<0. 0001) and counting (p<0. 0001) rituals, symmetry searching (p = 0.01) and ‘just right’ phenomena (p = 0. 001). Checking (p<0. 0001) and washing (p = 0. 001) rituals were highly specific for the ‘OCD-like’ group of RB in patients with GTS.

Finally, RB as ordering and hoarding rituals, impulsion phobias and intrusive images or thoughts could not be phenomenologically classified: there was not statistically significant difference between number of patients with criteria of tic or of compulsion for these types of behaviours (p = 0.3 for ordering, p = 0.5 for hoarding, p = 0.06 for phobias; and p = 1 for intrusive images and thoughts).

## Discussion

In accordance with previous reports [4], RB were a common symptom of GTS as 64.4% of our patients displayed one or more RB. Compared to patients without RB, those with RB had more severe and more frequent complex tics and SIBs, and were more frequently treated with neuroleptics. This is in line with recent reports showing segregated clusters of patients with GTS with different types of psychiatric manifestations [25] according to the characteristics of tics [26], [27]. It can be concluded that, independently of their phenomenological expression, the presence of RB is a hallmark of the severity of the syndrome.

Patients with GTS with RB could be categorised into three groups. In the first ‘tic-like’ group (24.1%), RB were performed without distress or anxiety and in response to an ‘urge to do’, that is in accordance with the definition of tics. The characteristic RB in this group were touching, counting, symmetry rituals and ‘just right’ phenomena. In the second, ‘OCD-like’ group of patients (20.4%), RB were performed to reduce anxiety, experienced as unwanted and senseless, and performed with the aim to protect the patients from real or potential negative events. Moderate to severe distress was observed when patients attempted to suppress or delay RB, a situation that corresponds to the criteria of compulsion such as those observed in anxious-type, non-tic related OCD. Characteristic RB in this group were checking and washing behaviours. The third, ‘mixed’ group (13.0%) could be identified in patients with both ‘tic-like’ and ‘OCD-like’ types of RB.

The clinical features of patients with GTS differed according to the type of RB observed. In the ‘OCD-like’ group compared to the ‘tic-like’ group (and to the non-RB patients), a higher score for complex tics and less successful socio-professional adaptation (higher score of the overall impairment item of the YGTSS and lower score of the GAF) was noted. In the mixed RB group compared to the ‘tic-like’ and ‘OCD-like’ groups, all clinical scores were higher with the exception of self-injurious behaviours and echophenomena. Taken together, these data suggest that ‘OCD-like’ RB differs from ‘tic-like’ RB, not only from a phenomenological point of view, but also in terms of treatment response and socio-professional adaptation. This contrasts with patients in the ‘tic-like’ RB group who had similar characteristics as the non-RB patients with rather limited impact on treatment response and socio-professional adaptation.

Several previous studies have examined the phenomenological characteristics of RB in patients with GTS (summarised in Table S2), many of which agree with our study insofar as symmetry, touching, counting and ‘just right’ phenomena were typical RB in patients with GTS. Also, in patients with GTS classified as having anxious-type OCD, washing and compulsions differed significantly from the ‘tic-like’ group. However, all of these studies have compared OCD and GTS patient groups and have not ascertained the prevalence of RB in GTS with regard to their ‘tic-like’ or ‘OCD-like’ characteristics, with the notable exception of Cath et al. [12], although a different etiology of ‘compulsive’ symptoms was hypothesised by several authors [11], [28]. Accordingly, most studies to date persist in classifying RB in patients with GTS as OCD. Current reviews still consider OCD as the major co-morbidity of GTS with rates up to 50% in the adult population [1], although the concept of ‘tic-like OCD’ has recently been put forward [29]. In contrast to the studies summarised in Table S3, only Shapiro and Shapiro [30] have clearly expressed the idea that some RB in patients with GTS may simply represent complex motor tics and termed them ‘impulsions’. However, we and other authors (Table S2) disagree with the low percentage of anxious-type OCD in patients with GTS as observed by Shapiro and Shapiro [30].

Obviously, the phenomenological distinction of RB in patients with GTS is important to consider, since the treatment of tics and OCD symptoms differs. Previous reports suggested that the presence of tics and GTS in OCD patients reduces the response of RB to symptomatic treatment, whether with SSRIs or cognitive-behavioural therapy [31], [32]. The distinction of two types of RB, either of the ‘tic-like’ or of the ‘OCD-like’ type, strongly suggest that the former should respond to neuroleptic treatments and habit reversal training and the latter to SSRIs or exposure-based cognitive behavioural therapy [33]. In addition, multiple studies of OCD symptoms suggested existence of four to six principal OCD-dimensions [8], [34], [35] that have been associated with different patterns of heritability and specific genetic polymorphisms [36], as well as with differential response to pharmacological and non-pharmacological treatments [37], [38]. Thus, distinction of RB into two different groups also suggests that the clinical expression of RB (‘tic-like’ or ‘OCD-like’) could result from the dysfunction of partially overlapping but distinct neuronal circuits. Both tics and OCD compulsions are considered to result from the dysfunction of cortico-striato-thalamo-cortical circuits [39]–[41]. In patients suffering from GTS without psychiatric co-morbidities, structural and functional neuroimaging studies as well as transcranial magnetic stimulation studies have shown dysfunction of premotor, sensorimotor, dorsal parietal, dorsolateral prefrontal and cingulated and insular cortical areas [42]–[44]. Similarly, in OCD patients with predominant ‘symmetry/ordering/counting’ behaviours but without tics, dysfunctions in motor, parietal and insular cortices have recently been described [45], [46]. Consequently, the ‘symmetry/ordering/counting’ dimension in OCD has not only close phenomenological characteristics to tics but may also share a similar neuronal basis. If so, this specific OCD dimension probably rather represents an integral part of GTS than being a distinct co-morbid condition.

In patients with OCD displaying washing and checking compulsions, neuroimaging studies have shown dysfunction of orbitofrontal, cingular and temporal cortices as well as of the caudate nucleus [46], [47]. Thus, ‘contamination/washing’ and ‘harm/checking’ OCD dimensions differ from tics not only by their phenomenological characteristics, but also in their underlying neuronal circuits and would therefore represent a true co-morbid condition. However, the caudate nucleus could play a prominent role in both disorders, since in two large MRI volumetric studies a diminished volume of the caudate nucleus was correlated with the presence of RB and persistence of tic and OCD symptoms into adulthood [48], [49]. In light of our results, we therefore stress the importance of a clear phenotypic characterisation of patients with GTS with regard to the nature of their RB (‘tic-like’, ‘OCD-like’ or both) in future neuroimaging studies.

### Limitations

First, if our sample is large, it is also heterogenous regarding age groups. Of note, 45/166 patients (27%) in our sample were 20 years or younger and may thus present a different phenotypic profile upon further aging. Second, no inferences on pathophysiology can be made based on descriptive cross-sectional research. Also, co-morbidity issues should be solved with the aid of family or twin studies, in which occurrence of tic-like or OCD-like behaviours is investigated in family members of probands with either GTS without OCD, GTS+OCD and OCD alone. These family studies [50], [51] have indicated that OCB/OCD and related repetitive behaviours in GTS patients are intrinsic to the pathology, although the debate is ongoing. Third, regarding therapeutic recommendations, it must be noted that OCD symptoms may also respond to neuroleptic treatment; however, pharmacological first line treatement of OCD remain SSRIs [52] and neuroleptics are currently rather used as augmentation treatment for treatment-resistant OCD [53]. Fourth, it can be argued that our results are induced by the method used since we have predefined classifications of the patients according to the symptoms observed (‘tic-like’ vs ‘OCD-like’) and then show that the same symptoms are distributed unevenly into these two categories. However, 6.3% of RB could neither be clearly attributed to the ‘tic-like’ nor the ‘OCD-like’ group and thus fall into a different category, albeit one that remains to be defined. The most important differential diagnosis in the domain of involuntary movements should be stereotypies which often co-exist with tics.

### Clinical implications

We suggest the importance of a precise semiological analysis of RB in patients with GTS, which may be particularly important for neurologists unfamiliar with the spectrum of OCD symptoms. We suggest that a substantial part of RB in patients with GTS are complex tics, as initially suggested by Shapiro and Shapiro [30] and warrant to be treated as such, either pharmacologically and/or by behavioural therapy. Conversely, neurologists facing OCD-like symptoms in GTS patients should seek treatment advice from their psychiatric colleagues, thus advocating a multidisciplinary approach in diagnosing and treating patients with GTS.

## Supporting Information

Table S1 General clinical and treatment characteristics of Gilles de la Tourette patients included in the study.
(0.04 MB DOC)

Table S2 Clinical and treatment characteristics of patients sub-groups with RB compared to patients without RB.
(0.05 MB DOC)

Table S3 Summary of previous studies investigating RB and/or OCD symptoms in patients with tics and/or GTS.
(0.05 MB DOC)

File S1 Semi-structured interview for the assessment of repetitive behaviours associated with tics.
(0.02 MB DOC)
