# Supplementary material for: Repetitive Behaviours in Patients with Gilles de la Tourette Syndrome: Tics, Compulsions, or Both?
Source: PLoS One. 2010 Sep 24;5(9):e12959. doi: 10.1371/journal.pone.0012959 (PMC2945770; doi:10.1371/journal.pone.0012959)
Supplement: Table S1 — General clinical and treatment characteristics of Gilles de la Tourette patients included in the study. (0.04 MB DOC) [file pone.0012959.s001.doc]

| Mean age (years old + SD) | 28.9 + 12.3 |
| --- | --- |
| Sex ratio (M/F) | 3.3/1 |
| ADHD (% of all patients) | 4.2 |
|  |  |
| ***Tics*** |  |
| Mean age of tics onset (years old + SD) | 9.0 + 5.0 |
| YGTSS total tics score ( / 50, points + SD) | 15.1 + 7.3 |
| YGTSS global score (/ 100, points + SD) | 41.7 + 16.8 |
| Self injurious behaviours ( % of all patients) | 33.7 |
| Constant tissue dammage (% of all SIBs) | 44.6 |
| Echophenomena (% of all patients) | 27.1 |
| Coprophenomena (% of all patients) | 33.1 |
| GAF (/ 100, points + SD) | 69.1 + 18.3 |
|  |  |
| ***Symptoms at onset (% of all patients)*** |  |
|  |  |
| Simple motor tics | 59.6 |
| Simple vocal tics | 30.1 |
| Complex tics | 10.2 |
|  |  |
| ***Family history ( % of all patients)*** |  |
| Transient tic disorders | 11.5 |
| Chronic tic disorders | 21.7 |
| GTS | 7.2 |
| OCD | 14.5 |
|  |  |
| ***Current medication (% of patients)*** |  |
| Without drug treatment | 34.3 |
| Neuroleptics | 45.8 |
| SSRIs | 23.5 |
| Benzodiazepines | 16.9 |
| Alpha2-adrenergic antagonists | 4.8 |
