# Supplementary material for: Repetitive Behaviours in Patients with Gilles de la Tourette Syndrome: Tics, Compulsions, or Both?
Source: PLoS One. 2010 Sep 24;5(9):e12959. doi: 10.1371/journal.pone.0012959 (PMC2945770; doi:10.1371/journal.pone.0012959)
Supplement: Table S2 — Clinical and treatment characteristics of patients sub-groups with RB compared to patients without RB. (0.05 MB DOC) [file pone.0012959.s002.doc]

| **Items** | **Groups** | | | |
| --- | --- | --- | --- | --- |
|  | **Without RB** | **Tic-like** | **OCD-like** | **Mixed group** |
|  |  |  |  |  |
| Number of patients (% of all patients) | 35.5 | 24.1 | 20.4 | 13.0 |
|  |  |  |  |  |
| Mean age (years old + SD) | 27.9 + 1.6 | 31.5 + 2.0 | 29.4 + 2 | 27.2 + 2.7 |
| Mean age of symptoms onset (years old + SD) | 7.7 + 0.5 | 8.4 + 0.6 | 8.5 + 0.7 | 10.0 + 0.9 |
|  |  |  |  |  |
| YGTSS, tic score (/50, points + SD) | 14.7 + 0.9 | 14.8 + 1.1 | 15.1 + 1.3 | 16.8 + 1.6 |
| YGTSS, overall imparement score (/50, points + SD) | 22.5 + 1.6 | 27.0 + 2.0 | 28.6 + 2.3 * | 29.5 + 2.8* |
| GAF (points + SD) | 72.7 + 2.8 | 67.7 + 3.5 | 62.6 + 3.8 * | 67.8 + 4.6 |
|  |  |  |  |  |
| Echophenomena score (points + SD) | 0.6 + 0.2 | 0.5 + 0.1 | 0.7 + 0.1 | 0.3 + 0.2 |
| Coprophenomena score (points + SD) | 0.4 + 0.1 | 0.8 + 0.2 | 0.7 + 0.2 | 0.9 + 0.2 |
|  |  |  |  |  |
| Complex tics score (points + SD) | 0.9 + 0.2 | 1.7 + 0.2 * | 2.2 + 0.2 *** | 2.0 + 0.3 ** |
|  |  |  |  |  |
| SIB frequency (%) | 23.7 | 43.6 | 39.4 | 38.1 |
|  |  |  |  |  |
| Neuroleptics treatement frequency (%) | 37.2 | 43.6* | 48.5* | 71.4* |
| SSRIs treatement frequency (%) | 11.9 | 20.5** | 33.3** | 38.1** |

SD – standard deviation, * - p < 0.05 ; ** - p < 0.01; *** - p < 0. 001
