# Supplementary material for: Repetitive Behaviours in Patients with Gilles de la Tourette Syndrome: Tics, Compulsions, or Both?
Source: PLoS One. 2010 Sep 24;5(9):e12959. doi: 10.1371/journal.pone.0012959 (PMC2945770; doi:10.1371/journal.pone.0012959)
Supplement: Table S3 — Summary of previous studies investigating RB and/or OCD symptoms in patients with tics and/or GTS. (0.05 MB DOC) [file pone.0012959.s003.doc]

| **Authors** | **Population** | **Methods** | **Main findings** | **Main interpretations** |
| --- | --- | --- | --- | --- |
| Frankel et al., 1986 | 63 GTS patients ; 11 OCD patients ; 41 normal controls (all age groups) | Questionnaire and LOI | GTS patients had more counting compulsions, doing things in a specified order, arranging items systematically, « touching » and obsessions about hurting oneself or others than the OCD and control groups. | Increased prevalence of OCD symptoms in GTS suggests a common neurobiological basis for GTS and OCD. |
| Pitman et al., 1987 | 16 GTS, 16 OCD and 16 normal controls (adults) | Yale Schedule for Tourette and Other Behavioural Syndrome-R, Maudsley Obsessive Compulsive Inventory, Eysenck Personality Inventory, Trait Portion of the STAI | More touching and symmetry behaviours in the GTS group. | Symptomatic overlap tends to blur GTS and OCD although some symptomatic poles tend to distinguish them. |
| Shapiro and Shapiro, 1988 | 666 consecutive GTS patients (all age groups) |  | Only 2-3% of GTS patients displayed symptoms corresponding OCD. | Repetitive behaviours in GTS patients usually correspond to complex motor tics and should be termed ‘impulsions’. |
| George et al., 1993 | 15 OCD/GTS patients, 10 OCD patients without GTS (adults) | Y-BOCS, Questionnaire, LOI | OCD/GTS patients had more violent, sexual, and symmetrical obsessions and more touching, blinking, counting, and self-damaging compulsions. OCD/GTS patients reported that their compulsions arose spontaneously. OCD patients had more obsessions concerning dirt or germs and more cleaning compulsions. OCD patients reported that their compulsions were frequently preceded by cognitions. | Phenomenologic differences between OCD and OCD/GTS which may reflect differential involvement of neurochemical and neuroanatomic pathways. |
| Baer, 1994 | 107 OCD patients (adults) | Y-BOCS, principal component factor analysis | "Symmetry/hoarding" on the Y-BOCS symptom shecklist was significantly related to a lifetime history of GTS or chronic tic disorder. |  |
| Leckman et al., 1994 | 177 OCD patients, 56 with tics, 121 without tics |  | Patients with tic-related OCD hade more religious, sexual and agressive obsessions as well as checking, counting, ordering, touching and hoarding compulsions than patients with non-tic-related OCD. No differences in « just right » phenomena between both groups. |  |
| Holzer et al., 1994 | 35 OCD and 35 OCD/tics patients (adults) | STOBS, Y-BOCS, HRSD | OCD/tics patients had more touching, rubbing, blinking and staring rituals. OCD patients has more cleaning rituals. | Compulsion subtype may help to differentiate between the two OCD subgroups. Nosologically, it remains to be determined whether certain compulsions in the OCD/tics groups may in fact correspond to complex motor tics. |
| Miguel et al., 1997 | 20 OCD, 21 GTS and 21 OCD/GTS patients (adults) | Semi-structured interview (USP-Harvard Repetitive Behaviours Interview), YGTSS, BAI, BDI | More cognitive and autonomic phenomena in the OCD group ; more sensory phenomena in the GTS group. The OCD/GTS group is intermediate. ‘Just right’ phenomena more frequent in the GTS group. No differences for contamination worries and cleaning compulsions between OCD and GTS patients. | Subjective experiences may be helpful in subtyping OCD and represent predictors of prognosis and treatment response. |
| Eapen et al., 1997 | 16 OCD patients and 16 GTS/OCB patients (all age groups) | Y-BOCS, CY-BOCS, NHIS, LOI | More dirt/germs/contamination obssessions in the GTS/OCB group ; more sexual obssessions in the GTS group. More washing/cleaning compulsions in the GTS/OCD group ; more symmetry, ‘just right’ and touching compulsions in the GTS group. | Different neuroanatomical and neurochemical substrates in GTS compared to GTS/OCB patients. |
| Müller et al., 1997 | 31 OCD, 18 GTS, 20 PD patients and 46 normal controls (adults) | MOCI, HZI-K, SDAS-LA | Significantly higher total scores in OCD and GTS patients on the MOCI and HZI-K scales compared to normal controls. Especially checking, ordering, counting and touching were prevalent in GTS patients, whereas cleaning and obsessive thoughts occured more frequently in OCD patients. Fearful obsessive thoughts is a discrimant item between OCD and GTS. | Specific patterns of obsessive-compulsive psychopathology may contribute to a more reliable differential diagnosis in OCD and GTS and help to avoid misdiagnosis of OCD in GTS patients. A common etiology of OCD and GTS is unlikely given the different spectrum of symptoms. Treatment (SSRIs and neuroleptics/α2-receptor antagonists) must be prescribed according to the prevailing symptomatology. |
| Zohar et al., 1997 | 40 adolescents with OCD, 15 with tics, 25 without tics | Semi-structured diagnostic interview, Y-BOCS | Patients with tics more prone to aggressive sexual images and obssessions than those without tics. | Symptom subtypes in OCD adolescents similar to those observed in OCD adults. |
| Petter et al., 1998 | 13 OCD/GTS patients, 13 OCD patients without GTS (adults) | Y-BOCS, YTGSS, ADHD checklist | OCD/GTS patients had more non-violent images, excessive concern with appearance, need for symmetry, touching, blinking, staring and counting compulsions than OCD patients without GTS. | Subtle but definite differences in symptomatology of patients with pure OCD compared to those with OCD/GTS. Putative differences in pathophysiology between the two groups, i.e., abnormalities in the serotonergic system in OCD patients and serotonergic and dopaminergic abnormalities in those with OCD/GTS and TS. Observations consistent with genetic heterogeneity within both OCD and GTS. |
| Cath et al., 2000 | 14 GTS/OCD, 18 GTS, 21 OCD/tics patients and 29 normal controls (adults) | Semi-structured diagnostic interview evaluating the goal-directedness and anxiety of symptoms (Leiden GTS/OCD interview), CIDI, MADRS, YBOCS, LOI, A-GAF, STAI | Obssessions were more time-comsuming and disabling than compulsions and impulsions. Highest rates of antidepressant medication in the tic-free OCD group. Patients with OCD/tics benefit less from monotherapy with SSRIs than tic-free OCD patients. | GTS/OCD is a more severe form of GTS. GTS/OCD is part of the GTS rather than the OCD spectrum. |
| Cath et al., 2001 | 14 GTS/OCD, 18 GTS, 21 OCD/tics patients and 29 normal controls (adults) | Semi-structured diagnostic interview evaluating the goal-directedness and anxiety of symptoms (Leiden GTS/OCD interview), CIDI, MADRS, YBOCS, LOI, A-GAF, STAI | GTS/OCD patients had more mental play, echophenomena, touching and more overall repetitive behaviours compared to the other groups. Tic-free OCD patients had more contamination worries and washing behaviours as well as more violent and sexual obssessions. OCD/tics patients were intermediate, but closer to tic-free OCD patients regarding their symptomatology. | GTS/OCD is a subform of GTS and not of OCD, or an entity of its own. Non-anxiety related « impulsions » probably reflect differences in underlying mechanisms between GTS and OCD/tics. |
| Hanna et al., 2002 | 60 OCD patients, 15 with a lifetime history of tics and 45 without tics (children and adolescents) | CY-BOCS, discriminant function analysis | Ordering, hoarding and washing compulsions were more common in OCD patients without tic history. | Tic-related OCD may be differentiated from non-tic-related OCD early in life by the presence/absence of certain compulsive symptoms. |
| Anholt et al., 2006 | 50 OCD, 19 OCD/tics, 18 GTS patients and 30 normal controls (adults) | SCID-I, YTGSS, OBQ-87, Padua-R Inventory, BAI, BDI | OCD patients exhibited higher OBQ-87 scores than GTS patients. Contamination fears discrimnate between OCD and GTS patients. However, no differences were found between OCD with or without tic patients on any of the OBQ-87 subscales. | Dysfunctional beliefs have no discriminative power with respect to OCD with or without tic patients and the direct relationship between types of obsesive-compulsive symptoms and specific dysfunctional beliefs is questionable. |
| Diniz et al., 2006 | 98 OCD, 31 OCD/patients with chronic motor or vocal tics (CVMT), 30 OCD/GTS patients | Semi-structured interview (USP-Harvard Repetitive Behaviour Interview), YGTSS, Y-BOCS | OCD/CMVT patients were similar to OCD/GTS patients regarding the frequency of intrusive sounds, repeating behaviors, counting and tic-like compulsions. For age at obsessive-compulsive symptom onset, sensory phenomena, number of comorbidities, frequency of somatic obsessions, bodily sensations and just-right perceptions, OCD/CMVT patients tended to be in between the other two groups. | There are qualitative and quantitative differences in the phenotypic expression of tic disorders in OCD patients, depending on whether the subject has GTS or CMVT. |

*Abbrevations*: ADHD = Attention-Deficit Hyperactivity Disorder ; A-GAF = DSM III-R Global Assessment of Functioning Scale-Adult Form ; BAI = Beck’s Anxiety Inventory ; BDI = Beck’s Depression Inventory ; CIDI = ; Composite International Diagnostic Interview ; GTS = Gilles de la Tourette Syndrome; HRSD = Hamilton Rating Scale for Depression ; HZI-K = Hamburg Obsessive-Compulsive Inventory ; LOI = Leyton Obsessional Inventory ; MADRS = Montgomery Asberg Depression Rating Scale ; MOCI = Maudsley Obsessive-Compulsive Inventory ; OBQ-87 = Obsessive-Compulsive Beliefs Questionnaire-87; OCB = ; NHIS = National Hospital Interview Schedule ; OCD = ; PD = Parkinson disease ; SADS-LA = Schedule for Affective Disorders and and Schizophrenia – Lifetime Version ; STAI = State-Trait Anxiety Inventory ; STBOS = Schedule for Tourette Syndrome and Other Behavioural Disorders ; Y-BOCS = Yale-Brown Obsessive Compulsive Scale; CY-BOCS = ; Children’s Yale-Brown Obsessive Compulsive Scale ; YTGSS = Yale Global Tic Severity Scale
