# Supplementary material for: Repetitive Behaviours in Patients with Gilles de la Tourette Syndrome: Tics, Compulsions, or Both?
Source: PLoS One. 2010 Sep 24;5(9):e12959. doi: 10.1371/journal.pone.0012959 (PMC2945770; doi:10.1371/journal.pone.0012959)
Supplement: File S1 — Semi-structured interview for the assessment of repetitive behaviours associated with tics. (0.02 MB DOC) [file pone.0012959.s004.doc]

**File S1**

**Semi-structured interview for the assessment of repetitive behaviours associated with tics**

The examination was focused on obsessions and rituals identified by the YBOCS symptom checklist.

The clinician must verify (in order to confirm that repetitive behaviours are not better explained by another psychiatric diagnosis):

• The egodystonic character of the symptoms.

• The degree of insight and conviction in relation to underlying beliefs.

• The absence of delusions, hallucinations, influence syndrome or mental automatisms.

• The independence of the symptoms in relation to a depressive syndrome.

• The independence of the symptoms in relation to a comorbid generalized anxiety disorder (feeling of guilt, ruminations on dreaded events which are possibly controlled by the subject, avoidance and/or rituals).

In case of presence of repetitive behaviours or mental acts which could be diagnosed as complex tics, the following questions have to be investigated to disentangle the diagnostic issue:

• Do behaviours aim to reduce an anxious tenseness?

• Do behaviours protect or secure the patient or others from real or potential risks?

• Are the repetitive behaviours dangerous physically for the patient or do they place the patient in a difficult position?

• What does the patient feel if she/he tries to do not execute the behaviour, or if she/he cannot execute the behaviour?

• What are the events dreaded by the patient at short and long term?

• Does the patient feel involved in or guilty for negative events that could occur?

**Conclusion**

The patient has repetitive behaviours:

• Which are certainly not tics (describe these symptoms).

• Which are probably not tics but for which a doubt persists (describe these symptoms).

• Which are certainly tics (describe these symptoms).
